# The primary outcomes and power calculations in clinical RCTs in urogynecology - need for improvement?

**DOI:** 10.1186/1745-6215-16-S1-P22

**Published:** 2015-05-29

**Authors:** Marianne Koch, Paul Riss, Wolfgang Umek, Engelbert Hanzal

**Affiliations:** 1 Department of Obstetrics and Gynecology, Medical University Vienna, Austria; 2 Karl Landsteiner Institute for Special Gynecology and Obstetrics, Vienna, Austria

## Background

Except for studies where composite outcomes are chosen, a randomized controlled trial must have a primary outcome parameter. The primary outcome must be unambiguous, reliably assessable and clinically relevant. The estimated difference between the primary outcome in the study and control group(s) is used for the power calculation to determine the number of subjects needed for the trial.

## Methods

We reviewed all RCTs published in three urogynecology journals (International Urogynecology Journal – IUJ), Neurourology and Urodynamics - NAU, Female Pelvic Medicine and Reconstructive Surgery - FPMRS) and three general gynecology journals (Obstetrics and Gynecology - GREEN, American Journal of Obstetrics and Gynecology – AJOG, and BJOG – an International Journal of Obstetrics and Gynecology) in the field of Urogynecology in the year 2013.

The journals were hand searched for clinical randomized controlled trials, and the following variables were noted: type of primary outcome, number of secondary outcomes, power calculation.

## Results

After excluding secondary analyses, a total of 34 randomized controlled trials were identified in the 6 journals in 2013 (IUJ 19, NAU 5, FPMRS 3, GREEN 6, AJOG 0, BJOG 1). 3/34 papers listed more than one primary outcome, while 8/34 papers did not list any secondary outcomes (Table 1).

**Table 1:** Number of primary and secondary outcomes

	N (range)
> 1 primary outcome	3/34

Secondary outcomes	26/34

mean secondary outcomes	3.5 (1-11)

The most common primary outcomes chosen by the investigators were **results of questionnaires** (n=8), and **POPQ** (pelvic organ prolapse quantification system) statements and **bladder diaries** (5 each).

Correct power calculations were done in **25/34** studies, and in **3** studies power calculation were reported in the methods section without giving the variable on which the power calculation was performed.

## Conclusion

We conclude that there is room for improvement for authors and journals in regard to identification of primary outcomes and to correct power calculation in RCTs.

